# Evaluation of an Effective Intradermal Foot-and-Mouth Disease Vaccine for Early Protection

**DOI:** 10.3390/vaccines14030263

**Published:** 2026-03-13

**Authors:** Dong-Wan Kim, Seo-Yong Lee, Tae-Jun Kim, Hyejin Kim, Ji-Hyeon Hwang, Sun Young Park, Young-Joon Ko, Yoon-Hee Lee, Jong-Hyeon Park, Sung-Han Park

**Affiliations:** Center for FMD Vaccine Research, Animal and Plant Quarantine Agency, 177, Hyeoksin 8-ro, Gimcheon-si 39660, Gyeongsangbuk-do, Republic of Koreajihyeonh87@korea.kr (J.-H.H.); koyoungjoon@korea.kr (Y.-J.K.); lyhee74@korea.kr (Y.-H.L.);

**Keywords:** foot-and-mouth disease, intradermal vaccination, early protection, FMDV serotype O, FMDV serotype A

## Abstract

**Background:** In South Korea, foot-and-mouth disease (FMD), a highly contagious viral infection that affects cloven-hoofed animals, has led to the implementation of a bivalent FMD vaccination program. The current FMD vaccination strategy involves intramuscular (IM) administration to the shoulder region of the swine. However, this method is associated with adverse reactions at injection sites. Our previous studies have demonstrated that intradermal (ID) vaccination eliminates these side effects while maintaining immunogenicity comparable to that of IM vaccination. This study aimed to assess the early immune response induced by ID vaccination and compare its protective ability against FMDV serotype O with that of a commercial IM vaccine recently used in South Korea. **Methods:** An ID FMD vaccine was evaluated using two adjuvants, ISA 207 (50%) and EMULSIGEN-D (15%). Virus neutralization (VN) titers and structural protein levels were measured to compare efficacy across groups. To assess the early protective efficacy of ID vaccination, viral challenge experiments were conducted at 7 and 14 days post-vaccination (dpv). **Results:** Swine vaccinated via the ID route exhibited no clinical symptoms at 14 dpv, indicating effective early protection against FMD (O/AS/SKR/2019). In addition, no side effects of FMD ID vaccination were observed. **Conclusions:** These results suggest that ID vaccination could serve as a viable alternative to conventional IM vaccination, which is frequently associated with adverse effects. Importantly, this study demonstrates that ID vaccination can provide effective early protection within 7–14 days post-vaccination, highlighting its potential utility for emergency outbreak control.

## 1. Introduction

The foot-and-mouth disease virus (FMDV) is a positive-sense, single-stranded RNA virus belonging to the genus Aphthovirus in the family Picornaviridae. Based on antigenic diversity, FMDV is classified into seven serotypes: O, A, Asia1, C, SAT1, SAT2, and SAT3. The virus infects a wide range of cloven-hoofed animals, including cattle, swine, sheep, and goats, and is characterized by its rapid transmission among susceptible hosts. As a result, outbreaks of FMDV can lead to substantial economic losses in the livestock sector. Foot-and-mouth disease (FMD) is considered one of the most economically important livestock diseases worldwide because it causes severe trade restrictions, decreased production efficiency, and the large-scale slaughter of infected and at-risk animals [1,2]. According to the World Organization for Animal Health (WOAH), annual economic losses due to FMD are estimated to exceed several billion US dollars, particularly in regions with high-density livestock farming [3].

In South Korea, a widespread serotype O outbreak occurred between November 2010 and April 2011, affecting both swine and cattle [4,5,6]. In response, the Korean government has implemented a mandatory vaccination policy for cloven-hoofed animals. The current FMD vaccination program relies primarily on oil-adjuvanted inactivated type O and A vaccines, which are administered intramuscularly (IM) [7]. However, the standard vaccination protocol, which mandates the use of a single-use syringe per pig, is often not strictly followed on farms, increasing the risk of secondary infections at the inoculation site. This can lead to adverse reactions such as abscess formation, granulomas, and necrosis [8,9]. The economic impacts of these side effects are of particular concern in South Korea, where pork shoulders are widely consumed.

Our previous study demonstrated that intradermal (ID) vaccination using a needle-free system (Pulse Needle-Free Systems, Lenexa, KS, USA) eliminated these adverse effects while maintaining an immunogenicity equivalent to that of IM vaccination [10]. Furthermore, ID vaccination has been shown to be more cost-effective than the conventional IM vaccine [11]. In addition to injection site reactions, IM vaccination has been reported to have limitations in inducing an optimal immune response. Studies have suggested that IM vaccines primarily activate systemic immunity while generating weaker mucosal immunity, which is critical for preventing viral entry into the primary infection site [12]. This limitation underscores the need for alternative vaccination strategies, such as ID administration, which target antigen-presenting cells (APCs) [13]. ID vaccines have been extensively evaluated for their efficacy against various pathogenic viruses including Ebola, hepatitis B, and canine influenza [14,15,16,17]. The success of ID vaccination is largely attributed to its targeted delivery to the epidermal layers, which are rich in antigen-presenting cells (such as dendritic cells, Langerhans cells, and macrophages), thereby enhancing immune response induction [13].

Additionally, previous studies have confirmed that ID vaccination leads to a rapid increase in antibody titers during the early stages of immunization [10]. Recent advancements in ID vaccination technology, including the development of needle-free delivery systems and microarray patches, have enhanced the feasibility of ID administration [18]. Studies have demonstrated that ID vaccination not only induces rapid seroconversion, but also enables dose-sparing effects, potentially reducing vaccine costs while maintaining efficacy.

To further explore the potential of ID vaccination against FMD, we evaluated the protective efficacy of an ID FMD vaccine using two different adjuvants, ISA 207 (50%) and Emulsigen-D (ED, 15%). This study aimed to assess the early immune response induced by ID vaccination at 7 and 14 days post-vaccination (dpv) and compare its protective ability against type O FMDV with that of a commercial IM vaccine recently used in South Korea.

## 2. Materials and Methods

### 2.1. Cells and Viruses

The LF-BK cells obtained from the Plum Island Animal Disease Center (Greenport, NY, USA) were employed for the viral neutralization test (VNT). These cells were maintained in Dulbecco’s modified Eagle’s medium (DMEM; Corning, NY, USA) supplemented with 10% fetal bovine serum (FBS; Gibco, Grand Island, NY, USA) and 1% penicillin-streptomycin (P/S; Gibco, Grand Island, NY, USA). For vaccine antigen production, baby hamster kidney cells (BHK-21) acquired from the American Type Culture Collection (ATCC, CCL-10) were used. The BHK-21 cells were cultured in Vento BHK200 serum-free medium (Merck, Darmstadt, Germany).

The FMDV strains O/ME-SA/PanAsia-2 (O PanAsia-2) and A22 Iraq/24/64 (A22 Iraq, GenBank accession no. AY593764.1) were utilized for vaccine antigen preparation as well as for the viral neutralization assay. Additionally, the strain O/Anseong/SKR/2019 (O AS, GenBank accession no. KU991734.1) was used to assess the protective efficacy of the vaccine in experimentally challenged swine.

### 2.2. Production of FMD Vaccine Antigens

The FMD vaccine used in this experiment contained the O PanAsia-2 and A22 Iraq 64 (A22 Iraq, GenBank accession number AY593764.1) strains. Antigen purification was performed as previously described [10], and the antigen payload (146S particles) was quantified prior to formulation, ensuring consistent antigen content per dose. The purified virus preparations were inactivated by adding 3 mM binary ethylenimine (Sigma-Aldrich, St. Louis, MO, USA) and incubating the mixture at 26 °C for 24 h in a shaking incubator [19]. Following inactivation, the virus suspension was precipitated by treatment with 7.5% polyethylene glycol 6000 and 0.5 M NaCl (Sigma-Aldrich) at 4 °C for 16 h. The mixture was subsequently centrifuged at 10,000× *g* for 30 min at 4 °C. The resulting pellet was further purified by sucrose density gradient ultra-centrifugation at 30,000 rpm for 4 h at 4 °C [20,21].

### 2.3. Vaccination and Viral Challenge

The vaccine contained 15 μg per dose of purified and binary ethylenimine (BEI)-inactivated O/ME-SA/PanAsia-2 and A22 Iraq/24/64 (A22 Iraq, GenBank accession number AY593764.1) antigens. The inactivated antigens were concentrated and purified by polyethylene glycol precipitation followed by sucrose density gradient ultracentrifugation, as previously described [9].

Each formulation was supplemented with 1% saponin and 10% aluminum hydroxide (Alum, Al(OH)_3_). Saponin (Saponin from Quillaja bark, Sigma-Aldrich, St. Louis, MO, USA) was used as a vaccine adjuvant. Alum (ALHYDROGEL^®^ Aluminium Hydroxide Gel Adjuvant, InvivoGen, San Diego, CA, USA) is an aluminum hydroxide gel suspension. Alum induces a Th2 response by improving the attraction and uptake of antigen by antigen-presenting cells (APCs).

ISA 207 and Emulsigen-D adjuvants were selected based on their distinct immunostimulatory properties and prior evidence of efficacy in FMD vaccine formulations. ISA 207 is a water-in-oil-in-water (W/O/W) emulsion adjuvant known to enhance humoral immunity and prolong antigen presentation, whereas Emulsigen-D is an oil-in-water (O/W) emulsion containing dimethyldioctadecylammonium bromide (DDA), which promotes rapid and robust immune activation. These adjuvants were chosen to compare early immune induction and protective efficacy following ID vaccination. For ID vaccination, two oil-based adjuvants with different emulsion types were evaluated: ISA 207 (50%; SEPPIC, Paris, France) and Emulsigen-D (15%; MVP Technologies, Omaha, NE, USA). Of the three adjuvant candidates, Emulsigen-DL90 (Emulsigen-DL90; MVP Technologies, Omaha, NE, USA) was excluded from the final candidate list.

Intradermal vaccination was carried out using the Pulse 250 needle-free injection system (Pulse Needle-Free Systems, Lenexa, KS, USA). Target animals in the Intradermal group were immunized via the intradermal route with a dose volume of 0.5 mL per vaccination. The IM group was administered a commercial vaccine at a dose of 2 mL/dose in the shoulder region. Both the experimental ID formulation and the commercial IM vaccine satisfy the ≥6 PD50 potency requirement per strain according to Korean FMD control standards. The commercial vaccine is a licensed bivalent formulation containing serotypes O and A, consistent with the experimental vaccine design. Because FMD vaccine efficacy is regulated based on PD50 potency rather than absolute antigen mass (μg), the comparison was designed to reflect field-representative vaccination conditions rather than strict antigen mass equivalence. Guinea pigs were vaccinated intradermally with 0.1 mL per dose.

All animal experiments were conducted in accordance with protocols approved by the Institutional Animal Care and Use Committee (IACUC) of the Animal and Plant Quarantine Agency, Republic of Korea (Approval no. 2023-750). Animals were housed in an animal biosafety level 3 (ABSL-3) facility at the Animal and Plant Quarantine Agency (APQA) and had free access to feed and water throughout the study. Clinically healthy, FMDV-seronegative swine (8–10 weeks old, weighing approximately 20–25 kg) were used in this study. Animals that showed negative antibody titers by FMDV SP ELISA were screened for the experiment and randomly assigned to the experimental groups before vaccination.

Blood samples were obtained for serological analysis. In guinea pig tests, blood samples were collected at 0, 3, 7, 14, 21, 35, 56, 84, 112, and 140 days post-vaccination (dpv). In comparative immunogenicity tests in swine, blood samples were collected at 0, 14, 28, 42, and 56 dpv. To evaluate the early protective efficacy of the vaccine in swine, blood samples were obtained from the target animals, at 0, 2, 4, 6, and 8 days post-challenge (dpc).

A commercial inactivated FMD vaccine currently used in South Korea was included as a positive control and administered intramuscularly according to the manufacturer’s instructions. An unvaccinated group was included as a negative control for comparison of immunological responses and clinical outcomes. Detailed descriptions are presented in Table 1 and Table 2.

Challenge experiments to evaluate protection against FMD virus were performed in an Animal Biosafety Level 3 (ABSL-3) facility at the Animal and Plant Quarantine Agency (APQA). The strain O/Anseong/SKR/2019 (O AS, GenBank accession no. KU991734.1) was used as the challenge virus. Animals were challenged at 7 and 14 dpv to assess early protective immunity. A detailed description of this process is provided in Table 3. Additionally, a commercial vaccination group was evaluated for early protection. The donor swine were inoculated with the challenge virus via the hoof at a dose of 1 × 10^6^ tissue culture infectious dose (TCID)/mL in a total volume of 0.1 mL. Following inoculation, the donor animals were co-housed with swine from the other experimental groups to enable virus transmission through direct contact. The donors were euthanized at 3 dpc. Clinical manifestations were evaluated using a seven-point scoring system, with one point assigned to each hoof (four points total) and one point each for lesions observed on the tongue, mouth, and nose. The assessment of clinical signs associated with FMD was performed according to previously described criteria [22].

### 2.4. Enzyme-Linked Immunosorbent Assay (ELISA)

The presence of antibodies against FMDV serotypes O and A was determined using the PrioCHECK FMDV Type O Antibody ELISA Kit (Thermo Fisher Scientific, Cleveland, OH, USA) and the VDPro FMDV Type A AB ELISA kit (MEDIAN Diagnostics, Chuncheon-si, Republic of Korea), respectively. Samples were considered positive when the percentage inhibition (PI) exceeded 50% for the serotype O ELISA kit, whereas an optical density (OD) value of ≥0.4 was used as the positivity threshold for the serotype A ELISA kit.

### 2.5. Viral Neutralization Test (VNT)

The viral neutralization test (VNT) was carried out in accordance with the protocol outlined in the World Organization for Animal Health (WOAH) Terrestrial Manual [23]. After separation, serum samples were heat-inactivated at 56 °C for 1 h. The serum samples were serially diluted 8, 16, 32, 64, 128, 256, 512, and 1024 times and mixed with 200 TCID50/0.05 mL of the O/AS/SKR/2019 virus strain. The final mixture (FMD virus and diluted serum) was reacted at 37 °C for 1 h, LF-BK cells were added, and the mixture was incubated at 37 °C for 72 h [23]. Antibody titers were calculated using the Spearman–Kärber method [24]. After 72 h, the cytopathic effect (CPE) was evaluated and neutralizing antibody titer was measured [25].

### 2.6. Real-Time Reverse Transcription Polymerase Chain Reaction (RT-PCR)

Viral RNA was isolated from serum and saliva samples using the Maxwell^®^ RSC RNA extraction kit (Promega Corporation, Madison, WI, USA). Detection of FMD virus RNA was performed using a one-step PrimeScript real-time RT-PCR kit (Bioneer, Daejeon, Republic of Korea) according to the manufacturer’s instructions. The assay targeted the FMDV 3D gene region using the following primers: 5′-GGA ACY GGG TTT TAY AAA CCT GTR AT-3′ and 5′-CCT CTC CTT TGC ACG CCG TGG GA-3′. The probe sequence (5′-ADC GCA GGT AAA GYG ATC TGT A-3′) was labeled with 6-FAM as the reporter dye and TAMRA as the quencher. Amplification and fluorescence detection were carried out using a CFX96 Touch™ Real-Time PCR Detection System version 3.1 program (Bio-Rad Laboratories, Hercules, CA, USA).

### 2.7. Statistical Analysis

Data are expressed as the mean ± standard error of the mean (SEM). Prior to parametric statistical analyses, data normality was assessed using the Shapiro–Wilk test. Statistical analyses were conducted using two-way analysis of variance (ANOVA), followed by Tukey’s multiple comparisons test implemented in Prism software (version 9; GraphPad Software, San Diego, CA, USA). Variance was calculated separately for each comparison. Significance levels were defined as * *p* < 0.05, ** *p* < 0.01, *** *p* < 0.001, and **** *p* < 0.0001; ns indicates not significant.

## 3. Results

### 3.1. Immunogenicity of Adjuvants for Use in the ID Vaccine

**Figure 1:**
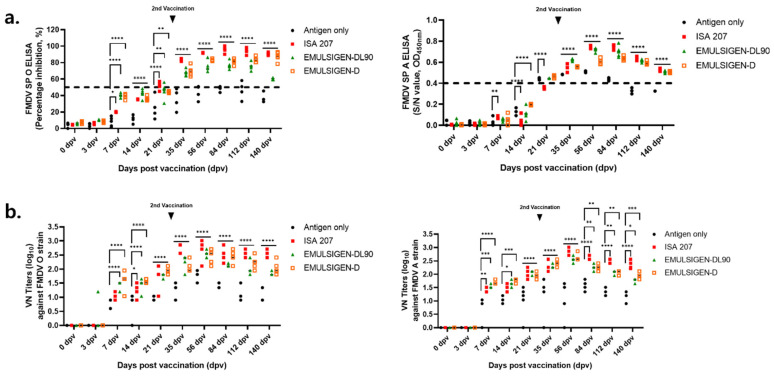
Comparison of the serological response after vaccination by adjuvant group in guinea pigs. Three adjuvants were used to evaluate long-term responses up to 140 days post-vaccination. Serum samples collected at 0, 3, 7, 14, 21, 35, 56, 84, 112, and 140 dpv were analyzed via VN assay and SP ELISA. PI values of FMDV type O and A SP ELISA in individuals. (**a**) PI and O.D. values of FMDV type O and A SP ELISA in individuals. (**b**) VN titers against the FMDV O and A strains. PI: percentage inhibition; VN: virus neutralization; SP: structure protein. Data represent the mean ± SEM; statistical analyses were performed using two-way ANOVA; *, *p* < 0.05; **, *p* < 0.01; ***, *p* < 0.001; and ****, *p* < 0.0001.

To compare the three selected adjuvants (ISA 207, ED, and EDL-90), their immunogenicity was tested in guinea pigs up to 140 dpv (Figure 1).

At 7 dpv, the ED and EDL-90 groups showed a PI value of >40%, and a positive value was confirmed at 35 dpv. However, the antibody titer in the EDL-90 group decreased after 84 dpv. The ISA 207 group showed a PI value of 20% in the early stage of vaccination, with the highest antibody titer being observed in the late stage. In guinea pigs, ED and EDL-90 showed high antibody titers in the early stages of vaccination, but EDL-90 showed a decline after 84 dpv, while ISA 207 had low initial antibody titers but maintained the highest antibody titers in the later stages.

**Figure 2:**
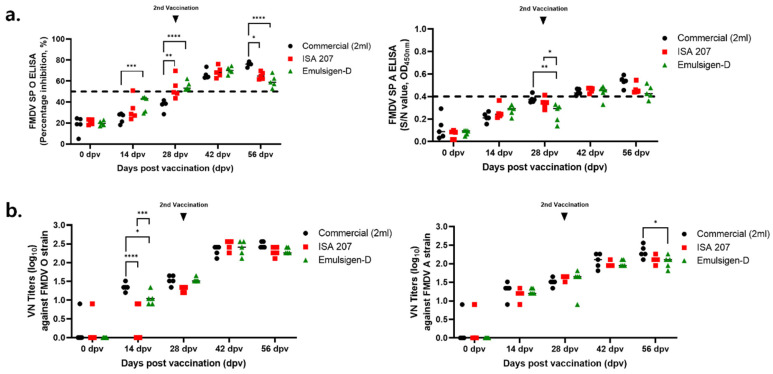
Comparison of the immune responses induced by two candidate adjuvants in swine. Both adjuvant formulations were monitored for up to 56 days post-vaccination (dpv). Serum samples collected at 0, 14, 28, 42, and 56 dpv were analyzed using a virus neutralization (VN) assay and structural protein (SP) ELISA. Individual percentage inhibition (PI) values for FMD virus serotypes O and A determined by SP ELISA are presented. (**a**) PI and O.D. values of FMDV type O and A SP ELISA in individuals. (**b**) VN titers against the FMDV O and A strains. PI: percentage inhibition; VN: virus neutralization; SP: structure protein. Data represent the mean ± SEM; statistical analyses were performed using two-way ANOVA; *, *p* < 0.05; **, *p* < 0.01; ***, *p* < 0.001; and ****, *p* < 0.0001.

The immunogenicity of the two adjuvants was compared with that of a commercial vaccine in swine, the target animal (Figure 2). Since the antibody titer of EDL-90 decreased in the later stages, it was excluded from the experiment. Up to 42 dpv, the three groups (ISA 207, ED and Commercial) showed similar antibody titers of >60%, and the VN titer was equivalent to 1:256 in all three groups.

Overall, both ISA 207 and Emulsigen-D induced comparable humoral immune responses to the commercial vaccine, supporting their suitability for ID vaccination.

### 3.2. Early Protective Efficacy of Intradermal Vaccination at 7 and 14 Days Post-Vaccination

#### 3.2.1. Serological Responses, Virus Detection, and Clinical Scores in 7 dpv Challenge Test

The animal experiments in Table 3 (7 and 14 dpv challenge tests) were conducted simultaneously, so that the negative and donor groups were the same.

**Figure 3:**
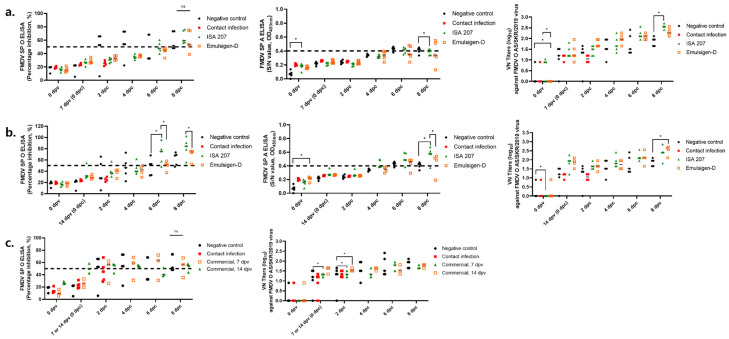
Serological responses determined by virus neutralization test (VNT) and structural protein (SP) ELISA following contact challenge with donor swine inoculated with the O/AS/SKR/2019 strain. Swine were challenged with the O/AS/SKR/2019 strain at 7 or 14 dpv. Serum samples collected at 0 and 7 or 14 dpv, as well as at 2, 4, 6, and 8 dpc, were analyzed using the virus neutralization (VN) assay. Neutralizing antibody titers against the O/AS/SKR/2019 strain are shown. Individual percentage inhibition (PI) values for FMD virus serotypes O and A determined by SP ELISA are also presented. (**a**) Serological analysis of the 7 dpv group. (**b**) Serological analysis of the 14 dpv group. (**c**) Serological analysis of the commercial vaccine group. PI: percentage inhibition; VN: virus neutralization; SP: structure protein; VNT: virus neutralization test. Data represent the mean ± SEM; statistical analyses were performed using two-way ANOVA; *, *p* < 0.05; **, *p* < 0.01; ***, *p* < 0.001; and ****, *p* < 0.0001.

Both types of SP-ELISA (types O and A) were negative on the first day of the viral challenge (0 dpc). At 6 dpc, the ISA 207 and ED groups showed antibody titers of 50% (Figure 3a). In both groups (ISA 207 and ED), average VN titers were ≥1:16 at 0 dpc and ≥1:100 at 6 dpc. At the end of the experiment, the ISA 207 group showed a VN titer of approximately 1:256 and the ED group showed a VN titer of 1:128 (Figure 3a).

**Figure 4:**
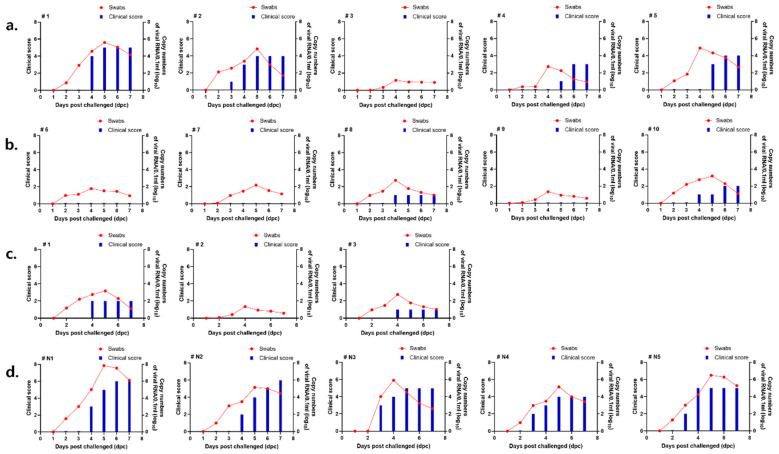
Clinical scores and FMD viral RNA levels in swine following challenge with donor inoculated with the O/AS/SKR/2019 strain. Swine were challenged with the O/AS/SKR/2019 strain at 7 dpv. Viral RNA levels in nasal swab samples were quantified by qRT-PCR from 1 to 7 dpc. The figure shows the clinical scores and FMD viral RNA levels for each experimental group. (**a**) Viremia and clinical scores of the ISA 207 adjuvant group. (**b**) Viremia and clinical scores of the Emulsigen-D adjuvant group. (**c**) Viremia and clinical scores of the commercial vaccine group. (**d**) Viremia and clinical scores of the negative group.

In the ISA 207 group, the virus was detected in four out of five swine, and the highest level was detected in a pig with a virus copy number (log 10) ranging from 1.115 to 5.607 (Figure 4a). In the ED group, the virus was detected in two out of five swine. In the ED group, viremia was relatively low (2.759) (Figure 4b).

Viral infection in swine was monitored by examining non-inoculated sites, including the hooves, mouth, and tongue, for the development of vesicular lesions. In the direct challenge group, clinical scores were assessed at 3 dpc, at which point a total score of four was recorded before the animals were euthanized.

Clinical symptoms were confirmed in the negative control group at 4 dpc, and the total score was calculated using 6 points at 6 dpc (Figure 4d).

In the ISA 207 group, clinical symptoms were confirmed in 4 out of 5 swine. Clinical signs were observed from 3 dpc, with a final clinical score of 5 (Figure 4a). In the ED group, symptoms were confirmed in 2 swine with a score of 2 (Figure 4b).

#### 3.2.2. Serological Responses, Virus Detection and Clinical Score in 14 dpv Challenge Test

In the 14 dpv challenge test, the ISA 207 group showed an average antibody titer of over 70% at 6 dpc. The ED group showed a positive antibody titer from 6 dpc and a titer of 60–70% at 8 dpc (Figure 3b). In both groups, the VN titer was as high as 1:100 on the day of virus challenge (Figure 3b). At the end of the experiment, ED and ISA 207 groups showed VN titers of 1:512 and approximately 1:256, respectively (Figure 3b).

**Figure 5:**
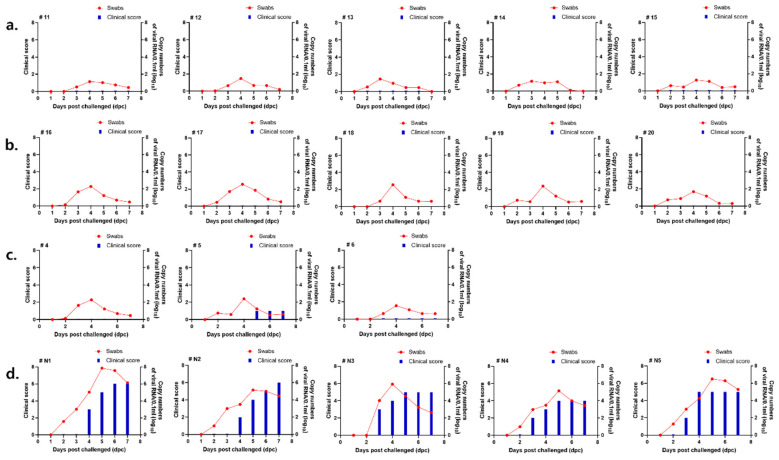
Clinical scores and FMD viral RNA levels in swine following challenge with donor inoculated with the O/AS/SKR/2019 strain. Swine were challenged with the O/AS/SKR/2019 strain at 14 dpv. Viral RNA levels in nasal swab samples were quantified by qRT-PCR from 1 to 7 dpc. The figure shows the clinical scores and FMD viral RNA levels for each experimental group. (**a**) Viremia and clinical scores of the ISA 207 adjuvant group. (**b**) Viremia and clinical scores of the Emulsigen-D adjuvant group. (**c**) Viremia and clinical scores of the commercial vaccine group. (**d**) Viremia and clinical scores of the negative group.

Low levels of viremia were detected in the ISA 207 group, and the viral copy number (log 10) was <1.479 (Figure 5a). In the ED group, the highest level of viremia was detected at 4 dpc, and the viral copy number (log 10) ranged from 2.286 to 2.563 (Figure 5b).

No clinical symptoms were observed in any of the intradermally vaccinated swine groups (Figure 5a,b). In the contact infection group, 6 points were measured at 3 dpc, after which the animals were sacrificed.

Taken together, clinical symptoms were observed in 2 (ED) and 4 (ISA 207) swine in the 7 dpv challenge test, and no clinical symptoms were observed in the 14 dpv challenge test. These findings demonstrate that ID vaccination, particularly with Emulsigen-D, provides improved early protection within 7–14 dpv compared with conventional IM vaccination.

### 3.3. Early Protection of Commercial Vaccine Against the FMDV Strain O/AS/SKR/2019

In the group challenged at 7 dpv, the type O SP ELISA showed a percentage inhibition (PI) value of approximately 20% at 0 dpc. In contrast, swine challenged at 14 dpv exhibited a PI value of about 50% at 0 dpc, which remained above the positive threshold throughout the experimental period (Figure 3c). The virus neutralization (VN) titer in the 14 dpv group reached ≥1:32 at 0 dpc. In both the 7 dpv and 14 dpv groups, the mean VN titers of vaccinated pigs ranged from 1:32 to 1:64 between 2 and 8 dpc (Figure 3c).

The 7 and 14 dpv groups showed viral copy numbers (log 10) ranging from 1.274 to 3.827 (Figure 4c) and from 1.329 to 3.175 (Figure 5c), respectively. Viremia was lower in the 14 dpv group than in the 7 dpv group. In the negative control group, the viral copy number (log 10) ranged from 4.017 to 7.854 at 4–5 dpc (Figure 4d and Figure 5d). The viral copy number (log 10) ranged from 3.999 to 6.499 in the contact infection group.

In the 7 dpv group, symptoms were confirmed in 2 out of 3 swine, and the score was relatively low at 2 points (Figure 4c). In the 14 dpv group, symptoms were confirmed in 1 out of 3 swine, with a score of 1 point (Figure 5c). The negative control group showed symptoms at 3 dpc, with a total score of 7 points at 6 dpc (Figure 4d and Figure 5d). In the contact infection group, 6 points were measured at 3 dpc, after which the animals were sacrificed. Brief information such as viremia and protection rate in the challenge test is described in Table 4.

## 4. Discussion

Vaccination is an effective strategy for controlling FMD, a serious infectious disease affecting the livestock industry. The Korean government has implemented a mandatory vaccination policy for cloven-hoofed animals to prevent FMD outbreaks [4]. However, current intramuscular (IM) vaccination strategies have limitations, including injection site reactions and suboptimal antigen presentation, owing to the relatively low presence of dendritic cells (DCs) in the muscle tissue [26,27].

Previous studies have reported that ID vaccination can overcome these drawbacks by efficiently targeting antigen-presenting cells (APCs) while reducing adverse reactions at the injection site [10]. Additionally, needle-free ID vaccination has been shown to elicit immune responses comparable to or better than those elicited by IM vaccines, providing an alternative route to enhance vaccine efficacy and safety [10].

In this study, we evaluated the early protective ability of an ID FMD vaccine using two different adjuvants, ISA 207 and ED, at 7 and 14 dpv. The results demonstrated that at 7 dpv, SP ELISA and VN titers were similar between the two groups; however, the protection rate differed significantly, with ISA 207 showing 20% protection and Emulsigen-D achieving 60% protection. At 14 dpv, both groups showed full protection (100%), indicating that ISA 207 may require a longer period to reach optimal immunogenicity.

ISA 207 and Emulsigen-D differ in their formulations and mechanisms of action. ISA 207 is a water-in-oil-in-water (W/O/W) emulsion-based adjuvant developed by SEPPIC, designed to enhance antigen presentation and promote the production of neutralizing antibodies. Previous studies have reported that ID administration of ISA 207 is highly effective in stimulating humoral immunity, making it suitable for long-term immune protection [28,29,30]. In contrast, Emulsigen-D is an oil-in-water (O/W) emulsion-based adjuvant developed by MVP Technologies and contains dimethyl dioctadecyl ammonium bromide (DDA), a potent immunostimulant [31]. Emulsigen-D is known to elicit a rapid and robust immune response, stimulating both humoral and cell-mediated immunity, which may explain its higher protection rate at 7 dpv compared to ISA 207. Studies have also suggested that Emulsigen-D can induce higher levels of humoral antibodies than aluminum-based adjuvants, contributing to faster immune activation.

ID vaccination offers several advantages over conventional IM vaccination, including reduced injection site reactions, lower antigen dosage requirements, and enhanced antigen presentation by APCs. IM vaccines often cause local injection site reactions, such as abscess formation and granulomas, which can negatively affect meat quality. By contrast, ID vaccination minimizes these adverse effects by delivering antigens more efficiently to APCs, leading to a stronger immune response and reduced tissue damage.

Furthermore, ID vaccination requires a lower antigen dose while maintaining equivalent or superior immunogenicity, making it a cost-effective alternative [32]. In addition to its immunological benefits, ID vaccination also provides practical advantages for large-scale livestock operations. Lower antigen dose requirements can significantly reduce vaccine production costs. Moreover, the use of needle-free injectors reduces cross-contamination risk and minimizes labor intensity, which is particularly beneficial in high-density farming environments.

This study has several strengths. First, the protective efficacy of intradermal vaccination was evaluated directly in the target species (swine) using a contact challenge model that closely reflects field transmission conditions. Second, two different adjuvant systems were compared to assess early immune induction following ID vaccination. However, several limitations should also be acknowledged. The number of animals included in each experimental group was relatively small, and this study primarily focused on early protection up to 14 days post-vaccination.

Although our study demonstrated strong early protection conferred by ID vaccination, further research is needed to evaluate the durability of immune responses beyond 14 dpv. Future studies should investigate strategies to enhance the durability of ID vaccine-induced immunity, such as booster vaccination schedules and novel adjuvant formulations. A comparative analysis of ID and IM vaccination strategies for long-term immunity will provide valuable insights for optimizing FMD vaccination protocols. To further validate these results, future studies should focus on long-term immunity beyond 14 dpv, field applicability in large-scale swine farms, and economic feasibility of switching from IM to ID vaccination. Additionally, considering that ID vaccination is a relatively new approach to FMD control, proper training of farm personnel using needle-free vaccination systems is essential to ensure consistent and effective vaccine administration. These findings highlight the importance of optimizing vaccination strategies to achieve rapid and effective protection during FMD outbreaks.

## 5. Conclusions

In summary, ID vaccination using ISA 207 or Emulsigen-D provided effective early protection against FMDV, with complete clinical protection observed at 14 dpv. Compared with conventional IM vaccination, ID administration reduced injection-site reactions while maintaining robust immunogenicity. These results support the potential utility of ID vaccination for emergency FMD control strategies.

## Figures and Tables

**Table 1:** Experimental designs of long-term immunogenicity by adjuvant candidates.

Group	No. of Guinea Pigs	Vaccine Strain	Serum Obtained at dpv
Antigen only, ID	5	O PA-2 + A22	0, 3, 7, 14, 21, 35, 56, 84, 112, 140
ISA 207, ID	5		
Emulsigen-DL90, ID	5		
Emulsigen-D, ID	5		

dpv, day post-vaccination; ID, intradermal.

**Table 2:** Experimental designs of vaccination by adjuvant candidates.

Group	No. of Swine	Vaccine Strain	Serum Obtained at dpv
Commercial vaccine, IM (2 mL)	5	Commercial	0, 14, 28, 42, 56
ISA 207, ID	5	O PA-2 + A22	
Emulsigen-D, ID	5		

dpv, day post-vaccination; ID, intradermal; IM, intramuscular.

**Table 3:** Experimental designs of FMD vaccination (7 dpv) against the FMD virus.

Group	No. of Swine	Vaccine Strain	Challenge Virus	Serum Obtained at dpc	Swab Obtained at dpc
Negative (Unvaccinated)	5	-	O/AS/SKR/2019	−7, 0, 2, 4, 6, 8	1–7
ISA 207, ID (7 dpv)	5	O PA-2 + A22			
Emulsigen-D, ID (7 dpv)	5				
Commercial, IM (7 dpv)	3	Commercial			
ISA 207, ID (14 dpv)	5	O PA-2 + A22	O/AS/SKR/2019	−14, 0, 2, 4, 6, 8	1–7
Emulsigen-D, ID (14 dpv)	5				
Commercial, IM (14 dpv)	3	Commercial			

dpv, day post-vaccination; dpc, day post-challenge; ID, intradermal; IM, intramuscular.

**Table 4:** Summary of early immunological and protective outcomes following FMDV challenge.

Group	VN Titer at 0 dpv	Protection Rate (%)	Peak Viral Load (log 10)
ISA 207 (ID, 7 dpv)	≥1:16	20%	1.115–5.607
ED (ID, 7 dpv)	≥1:16	60%	≤2.759
Commercial (IM, 7 dpv)	Low	33%	1.274–3.827
ISA 207 (ID, 14 dpv)	≥1:100	100%	<1.479
ED (ID, 14 dpv)	≥1:100	100%	2.286–2.563
Commercial (IM, 14 dpv)	≥1:32	67%	1.329–3.175
Unvaccinated	Negative	0%	4.017–7.854

dpv, day post-vaccination; ID, intradermal; IM, intramuscular; VN, virus neutralization.

## Data Availability

The data presented in this study are available upon request from the corresponding author. The data are not publicly available because of institutional policies.

## Abbreviations

The following abbreviations are used in this manuscript:

FMDV	Foot-and-mouth disease virus
IM	Intramuscular
ID	Intradermal
VN	Virus neutralization
APCs	Antigen-presenting cells
VNT	Viral neutralization test
ED	Emulsigen-D
dpv	Day post-vaccination
dpc	Day post-challenge
TCID	Tissue culture infectious dose
ELISA	Enzyme-linked immunosorbent assay
RT-PCR	Real-time reverse transcription polymerase chain reaction
PI	Percentage inhibition
SP	Structure protein
